# Psychosocial Distress in Women With Breast Cancer and Their Partners and Its Impact on Supportive Care Needs in Partners

**DOI:** 10.3389/fpsyg.2020.564079

**Published:** 2020-09-23

**Authors:** Ute Goerling, Corinna Bergelt, Volkmar Müller, Anja Mehnert-Theuerkauf

**Affiliations:** ^1^Charité Comprehensive Cancer Center, Berlin Institute of Health, Charité – Universitätsmedizin Berlin, Corporate Member of Freie Universität Berlin, Humboldt-Universität zu Berlin, Berlin, Germany; ^2^Department of Medical Psychology, University Medical Center Hamburg-Eppendorf, Hamburg, Germany; ^3^Department of Gynecology, University Medical Center Hamburg-Eppendorf, Hamburg, Germany; ^4^Department of Medical Psychology and Medical Sociology, University Medical Center Leipzig, Leipzig, Germany

**Keywords:** breast cancer, partner, distress, needs, psycho-oncology

## Abstract

**Objectives:**

While both patients and informal caregivers report high levels of cancer-related distress, supportive care needs of relatives are often not taken into account and little is known about mutual perception of distress within couples. Therefore, we aimed to investigate distress in female patients with breast cancer and their male partners as well as supportive care needs in partners.

**Methods:**

In this cross-sectional study, we recruited women with breast cancer during primary cancer care and their male partners, obtained information on mental distress and supportive care needs through visual analog scales for four mood domains and the Short Form of Supportive Care Needs Survey (SCNS-SF34).

**Results:**

Among 250 eligible patients with breast cancer, 102 patients (40.8%) and their male partners participated. Partners reported higher levels of distress (*p* = 0.02), whereas patients (self-assessment) indicated stronger needs for help (*p* < 0.001). Men with higher levels of distress were younger (*p* < 0.001), and reported a shorter relationship duration (*p* = 0.001) compared to partners with lower distress. Partners overestimated distress, anxiety, depression, and need for help in the patient. Patients overestimated partners need for help. The majority of partners (78%) reported at least one unmet need, most frequently related to the health system and information domain.

**Conclusion:**

A systematic distress and needs assessment for women with breast cancer and their male partners is mandatory. The provision of optimal supportive care depends on protocols that include not only psychosocial care for patients but also procedures for managing distress and needs for partners including individual and couple-based interventions.

## Introduction

It is well-known that breast cancer and its treatment have a debilitating effect on patients suffering from health restrictions such as lymphedema, pain, early menopause, and sexual problems (Baucom et al., 2006), as well as from wide-ranging psychosocial consequences like anxiety, depression, and self-image concerns (Bloom, 2002; Mitchell et al., 2011; Ng et al., 2011). An extensive review on unmet needs of women with breast cancer concludes that the greatest need is in the area of health system/information and psychological topics (Fiszer et al., 2014).

Cancer and its treatments also have an impact on the family environment. Several studies yielded to examine this issue including mood disturbance and mental burden of family members (Pitceathly and Maguire, 2003; Drabe et al., 2013; Williams et al., 2013; Schrank et al., 2016). Kayser et al. (2007) postulated cancer as a “we-disease.” Patients and caregivers show clinically relevant cancer-related distress and sometimes caregivers report even higher levels of anxiety than patients (Northouse et al., 2012; Feiten et al., 2013; Gröpper et al., 2016). In contrast, another study found that 35.7% of patients with breast cancer and 16.1% of partners report moderate to severe distress (Dumitra et al., 2018). A comparison of spouses and other social network members of women with breast cancer and prostate cancer patients revealed high depression levels in spouses (Segrin and Badger, 2010). Studies on gender and role (patient vs. partner) in couples dealing with cancer show that women in both roles report higher burden than males and that women and male partners report lower quality of life compared to the general population (Bergelt et al., 2008; Drabe et al., 2016). Distress levels and quality of life do not differ between female patients and female partners, and both groups report higher distress and lower quality of life than unaffected controls, while male partners report higher distress levels than male patients (Hagedoorn et al., 2000).

The majority (60%) of women with breast cancer regards their partner as their main source of emotional support (Sandgren et al., 2004). However, patients with breast cancer frequently report that they do not talk about their feelings with the family (Faller, 1998). Insufficient communication about perceived distress, anxiety and related issues can lead to misperception of burden in both partners, which can affect the couple’s relationship and further adaptive coping efforts. In palliative situations, family caregivers overestimate symptoms in patients (Oechsle et al., 2013b) and increased anxiety in family caregivers is associated with a discrepancy in the patients’ symptom evaluation in terminally ill patients (Oechsle et al., 2013a).

Both patients and partners are facing major challenges in diagnosis and treatment. Dyadic coping is characterized by interaction between stress signals in one partner and the coping response of the other. According to Bodenmann’s systemic-transactional theory (Bodenmann, 1997) coping is a stress management process in which one partner either ignores or reacts to the other partner’s stress signals in order to maintain a level of stability at the individual level on one hand and at the dyadic level on the other hand. Couples react as an emotional system and not as individuals (Hagedoorn et al., 2008). The partners’ distress is significantly related to lower relationship satisfaction (Pankrath et al., 2018). Higher distress in partners might adversely influence dyadic coping processes and have a negative impact on patients’ and partners’ quality of life and the relationship quality (Segrin, 2005; Kayser et al., 2007; Brusilovskiy et al., 2009; Robbins et al., 2014). A study of 42 couples in whom the male partner suffer from prostate cancer highlighted the association between perception of negative coping in each other with a higher psychological burden (Regan et al., 2014). These results are complemented by a study in patients with metastatic breast cancer and their partners which has shown that positive joint coping strategies lead to lower stress levels and better adaptation to the situation for both partners (Badr et al., 2010). Open and constructive communication seems to improve dyadic coping (Brandão et al., 2017).

In Germany, according to guidelines of the German Cancer Society, the German Cancer Aid and the Working Groups of the Scientific Medical Societies for the treatment of breast cancer (2020) and also according to the guideline on psycho-oncological diagnostics and care of cancer patients (2014), a routine distress screening in patients is mandatory, which entails that psycho-oncological care often focuses on supporting the patient while distress in relatives might remain undetected.

Studies on the mutual assessments of distress in women with breast cancer and their male partners and the resulting supportive care needs are rare. The general aim of this study was to increase this knowledge in order to allow for the development of improved support strategies for patients and their partners.

Therefore, the study focused on the following objectives and hypotheses:

(1) How do female patients and their male partners evaluate their own distress levels (self-assessment)? We hypothesize that patients and partners do not differ significantly in this regard.
(2) How do male partners evaluate the distress level of female patients and vice versa (other reported assessment)? We hypothesize that partners overestimate the mental burden of the patients, and that the patients underestimate the mental burden of their partners.
(3) What supportive care needs do male partners of patients with breast cancer report?

## Materials and Methods

### Study Design

In this cross-sectional study, we recruited women with breast cancer and their male partners over a 2-year period at the Breast Cancer Center, University Medical Center Hamburg, Germany. Patients were eligible if they were (i) diagnosed with a malignant tumor of the breast according to the medical record and/or their treating physician, (ii) 18 years or older, (iii) living in a heterosexual relationship, (iv) able to speak and read German and (v) able to give informed consent for study participation. Eligible patients were consecutively recruited by a trained study research assistant during outpatient chemotherapy treatment and were asked to complete a set of validated questionnaires. Each female patient asked her male partner if he was interested to participate in the study. Upon approval, the partner received a questionnaire in a closed envelope. Both patients and partners had to provide written informed consent and received prepaid envelopes to return the completed questionnaires. A color differentiation and unambiguous labeling ensured that the questionnaires were clearly assigned; (self-assessment vs. other reported assessment). The study participants filled out the questionnaire at home. Completing the questionnaires took about 20 min for each participant (patient and partner). A single reminder was sent out after 4 weeks.

The study was carried out in accordance with the Declaration of Helsinki and the data privacy protection laws and approved by the local ethics committee (Ethics Board Hamburg Number PV4560).

### Measures

Medical data from the patients were obtained from the electronic hospital information system including location of the tumor, time of first diagnosis, time of current diagnosis (if recurrence or second tumor), TNM-classification, current treatment as well as relevant somatic comorbidities. Partners completed a questionnaire: items included age, relationship to the patient, number of children, level of education, employment status and monthly household income.

Patients were asked to assess how burdened they felt themselves due to their cancer (self-assessment). Furthermore, they were asked to estimate the level of burden in their partner (other reported assessment). Partners were asked to assess how burdened they felt because of the cancer (self-assessment) and how they assessed the burden of the patient (other reported assessment). Partners were also asked about their support needs.

#### Emotion Thermometers (ET)

To assess the mental burden we used the Emotion Thermometer (Mitchell et al., 2010). This visual analog scale covers for four mood domains: distress, anxiety, depression and anger. We additionally included need for help as a further domain. Participants were asked to rate every domain from 0 (“not at all or no help necessary”) to 10 (“extreme”). Following literature recommendations, we used a cut-off of 5 in the distress domain (Mehnert et al., 2006; Hinz et al., 2019).

#### Supportive Care Needs Survey – Short Form 34

The German version of the Supportive Care Needs Survey – Short Form 34 (SCNS-SF-34) measures participants’ perceived kind and level of need for support in five domains: health system and information, psychological, physical and daily living, patient care and support, and sexuality needs (Lehmann et al., 2012). If in need of support in a domain, they are asked to rate level of the need on a 5-point Likert scale (1 = no need, 2 = no need, satisfied, 3 = low need, 4 = moderate need, 5 = high need). For each scale, sum scores were calculated (between 0 and 100). High scores indicate high supportive care needs. Furthermore, the answers were dichotomized, i.e., answer alternatives 1 or 2 mean no need for support and answer alternatives 3–5 mean need for support. The reliability of the five scales is high with Cronbach’s alpha ranging from 0.82 to 0.95 (Nunnally, 1978).

### Statistical Analyses

Sociodemographic and medical characteristics of the samples were analyzed by descriptive statistics. Group differences regarding self-assessment and assessment of the partner for distress (other reported assessment) were analyzed by using *t*-test for independent samples. We analyzed associations between categorical variables using chi-square test. The association between the assessment of the partner’s burden and self-assessed mental burden was analyzed by Pearson’s correlation. Linear regression analyses were conducted to analyze association in mutual assessment. Therefore, we included age, marital status, children, school qualification, living together, and duration of relationship.

Supportive care needs were analyzed according to the manual of the SCNS-SF34 by calculating means and frequencies. The association between distress and other supportive care needs was analyzed by Pearson’s correlation. The level of significance for all tests was defined as *p* = 0.05. Statistical analyses were conducted using SPSS statistical software for windows version 25.

## Results

### Sample Characteristics

**FIGURE 1:**
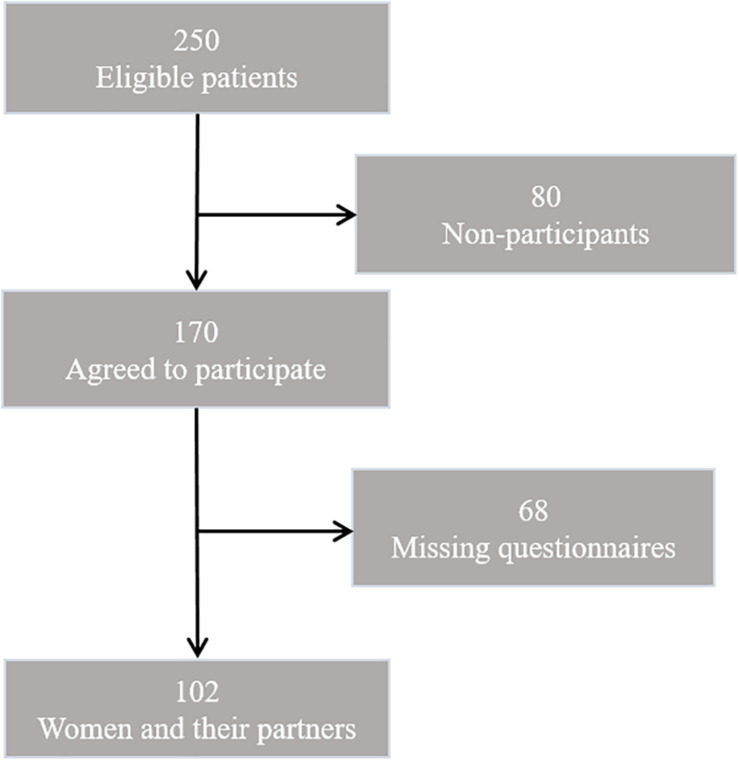
Study sample recruitment.

Out of 250 eligible female patients with breast cancer, 102 female patients (40.8%) and their male partners agreed to participate, and provided complete data (Figure 1). The reasons given for non-participation were excessive physical or mental burden, lack of interest, or the certainty that the partner would not participate. The mean age of the patients was 54 years (SD = 13.2; range 26–81) and of their partners 56 years (*SD* = 13.5; range 26–81).

Table 1 summarizes demography of the total sample as well as the medical characteristics of patients with breast cancer.

**TABLE 1:** Sample characteristics.

**Demography**	**Patients (*N* = 102)**	**Partners (*N* = 102)**
Age (years): Mean, SD^†^, min-max	54 (13.2, 26–81)	56 (13.5, 29–84)
Sex	100% female	100% male
Marital status: n (%)		
Unmarried		20 (19.6)
Married		74 (72.5)
Divorced		6 (5.9)
Widowed		2 (2.0)
Children: n (%)		62 (60.8)
Living together: n (%)		93 (91.2)
Duration of relationship (years): Mean, SD^†^, min-max		25 (16.3, 0.3–63)
Medical characteristics		
Disease situation: n (%)		
Primary tumor	62 (60.8)	
Secondary tumor	7 (6.9)	
Relapse	33 (32.4)	
Time since diagnosis (years): Mean, SD^†^, min-max	3.8 (5.7, 1–24.4)	
UICC^‡^ cancer stage: n (%)		
I	23 (22.5)	
II	27 (26.5)	
III	12 (11.8)	
IV	40 (39.2)	
Treatment option: n (%)		
Curative	62 (60.8)	
Palliative	40 (39.2)	
Surgery: n (%)		
Yes	60 (58.8)	
No	42 (41.2)	
Kind of surgery: n (%)		
Breast conserving surgery	43 (71.7)	
Mastectomy	17 (28.3)	
Current treatment: n (%)		
Chemotherapy	84 (82.4)	
Radiotherapy	3 (2.9)	
Anti-hormonal therapy	12 (11.8)	
Other	36 (35.2)	

*^†^SD, Standard deviation; ^‡^UICC, Union internationale contre le cancer.*

### Comparison of Distress Between Female Patients and Male Partners (Self-Assessments)

Patients and partners reported similar levels of anxiety, depression and anger, while partners reported significantly higher distress levels than patients. In contrast, patients reported more need for help than partners (Table 2).

**TABLE 2:** Mental burden in female patients with breast cancer and their male partners (self-assessment).

	**Female patients (self-assessment) (*N* = 102) Mean (*SD*^†^)**	**Male partners (self-assessment) (*N* = 102) Mean (*SD*^†^)**	**Difference**		
	**Mean (*SD*^†^)**	**Mean (*SD*^†^)**	**Mean**	**(*SD*^†^)**	***p***
Distress^+^	4.21 (2.81)	5.02 (2.91)	−0.81	3.48	0.02
Anxiety^+^	4.63 (3.07)	3.90 (3.87)	0.73	3.93	0.06
Depression^+^	2.62 (3.04)	2.04 (2.73)	0.58	3.70	0.12
Anger^+^	3.18 (2.99)	2.67 (2.93)	0.55	3.27	0.10
Need for help^+^	4.00 (2.97)	2.50 (2.69)	1.51	3.44	**<0.00**

*^†^SD, Standard deviation. ^+^Measured with the Emotion Thermometer. 0 = low distress/need for help, 10 = extreme distress/need for help.*

47% of the patients and 62% of the partners reported clinically relevant distress levels above cut-off (*p* = 0.043). Highly distressed partners are significantly younger (51.2 years vs. 62.1 years; *p* < 0.001), and reported a shorter relationship duration (19.6 years vs. 31.5 years; *p* = 0.001) than less distressed partners, but do not differ to them with regard to having children (*p* = 0.14), school education (*p* = 0.25) or cohabitation (*p* = 0.35).

### Mutual Assessment of Distress in Patients and Partners (Other Reported Assessment)

Male partners overestimated distress, anxiety, depression, and need for help in the patient, compared to the patients’ self-assessment, while anger was evaluated similarly to the patients’ report. The evaluation for the patient by her male partners (other reported assessment) was significantly associated to the self-perceived mental burden in the male partner (Table 3).

**TABLE 3:** Associations between male partners’ assessment of female patients’ mental burden (other reported assessment) and needs and patients self-assessment of mental burden and needs (Pearson’s correlation).

				**Self-assessment of partners**				
	**Patients self-assessment (*N* = 102) Mean (*SD*^†^)**	**Partner’s assessment of the patient (other reported assessment) (*N* = 102) Mean (*SD*^†^)**	***p*-value**	**Distress in partners**	**Anxiety in partners**	**Depression in partners**	**Anger in partners**	**Need for help in partners**
Distress^+^	4.21 (2.81)	5.48 (2.98)	**<0.001**	0.354**				
Anxiety^+^	4.63 (3.07)	6.18 (3.07)	**<0.001**	0.247*	0.249*			
Depression^+^	2.62 (3.04)	3.73 (3.34	**0.002**	0.232*	0.255*	0.282*		
Anger^+^	3.18 (2.99)	3.23 (3.15)	0.890	0.307**	0.341**	0.316**	0.306**	
Need for help^+^	4.00 (2.97)	5.32 (2.99)	**<0.001**	0.176	0.386**	0.195	0.163	0.235*

**The correlation is significant at a level of p = 0.01 (both sides). *The correlation is significant at a level of p = 0.05 (both sides). ^†^SD, Standard deviation. ^+^Measured with the Emotion Thermometer. 0 low distress/need for help – 10 extreme distress/need for help.

Age, marital status, children, school qualification, living together, and duration of relationship were not significantly associated with the partner’s overestimation of distress, anxiety, depression, and need for help in the patient (other reported assessment). There were no significant differences between patients’ assessment of the partner’s situation and partners self-assessment with regard of distress (5.05 vs. 5.02, *p* = 0.90), anxiety (4.00 vs. 3.90, *p* = 0.72), depression (1.78 vs. 2.04, *p* = 0.22) and anger (2.40 vs. 2.67, *p* = 0.35). Patients did, however, overestimate need for help in their partners (3.08 vs. 2.50; *p* = 0.03).

### Supportive Care Needs in Partners

The majority of partners (78%) reported at least one unmet need (*M* = 14, *SD* = 11.6). All supportive care needs were significantly positively associated to higher levels of anxiety, depression and distress (see Table 4).

**TABLE 4:** Association of supportive care needs in male partners and mental burden in male partners (self-assessment) of female patients with breast cancer (Pearson’s correlation).

**Supportive care needs^++^**	**Anxiety^+^**	**Depression^+^**	**Distress^+^**
Health system and information	0.40**	0.32**	0.27**
Psychological needs	0.69**	0.56**	0.47**
Sexuality needs	0.49**	0.51**	0.43**
Physical and daily living needs	0.57**	0.50**	0.44**
Patient care and support	0.42**	0.36**	0.30**

**The correlation is significant at a level of p = 0.01 (both sides). ^+^Measured with the Emotion Thermometer; ^++^measured with the Supportive Care Needs Survey.

The highest rated needs according to the SCNS-SF-34 in male partners are in the domain health system and information (*M* = 38.9; *SD* = 34.9), followed by psychological needs (*M* = 34.7; *SD* = 31.7), sexuality needs (*M* = 29.7; *SD* = 32.1), physical and daily living needs (*M* = 23.5; *SD* = 22.4), and needs in patient care and support (*M* = 20.2; *SD* = 25.2).

## Discussion

This study examined psychosocial distress in women with breast cancer and their male partners and the impact on supportive care needs in partners. As hypothesized, we found that female patients and their male partners did not differ in self-assessment with regard to anxiety levels, depressive symptoms and anger. These findings support the results of other studies (Hodges et al., 2005; Rosenberger et al., 2012). The relatively high anxiety levels in this sample might be treatment-related, since most of the women (82%) were recruited during chemotherapy. A review of studies conducted between 1990 and 2010 also showed high anxiety levels in patients during chemotherapy (Lim et al., 2011). Interestingly, in our study partners reported higher distress than patients, which is in line with another study, were primary caregivers were more distressed than the respective patients (Sklenarova et al., 2015). Also previous studies found a higher global burden in spouses (Hasson-Ohayon et al., 2010; Segrin and Badger, 2010). Further, meta-analyses showed, that male partners have reported more distress when the women is the patient in the couple (Hagedoorn et al., 2008). However, some other studies also reported higher distress in women than in men regardless of their role as patient or caregiver (Hagedoorn et al., 2008). Our results do not necessarily contradict these findings. Our results rather indicate that the diagnosis and treatment of breast cancer in their wives are extremely burdening for male partners. A study on 96 dyads suggested that the within-dyad influence runs mostly from partners anxiety to the anxiety of women with breast cancer (Segrin et al., 2007).

Even though partners in our sample perceived higher distress levels than female patients, they reported fewer needs for help. This might possibly be explained by the treatment situation, in which women are physically impaired and therefore express more needs for help. Further, this finding could be gender-related: men might not perceive or admit supportive care needs due to their gender role perceptions. In the literature similar results are reported: women both reported more request for help (Merckaert et al., 2010) and accepted more help than men (Curry et al., 2002). Male partners also provide practical support for the patient and might thus neglect their own needs in this situation.

In our study, patients were asked, after having assessed their own burden (self-assessment), to assess their partner’s burden (other reported assessment) caused by their wives cancer disease. Accordingly, the partners were asked to assess their own burden first (self-assessment) and then the burden of the patient (other reported assessment). Our hypothesis that women overestimate mental burden distress in their partner (other reported assessment) was only confirmed with regard to need for help. Considering the sequence of questions, this might be an expression or projection for their own need and wish for help. This assumption matches and supports the clinical experience that women often wish for help for their partners. However, the mutual evaluation of female patients by male partners (other reported assessment) revealed an overestimation of distress, anxiety, and depression as well as for need for help. This was associated with high distress and mental burden in partners. These results are in the line with another study reporting that family caregivers of patients receiving chemotherapy typically overestimate cancer patients’ symptom burden and suffer from considerable mental burden themselves (Silveira et al., 2010). Our study also investigated supportive care needs in partners and has shown that partners of women with breast cancer report high levels of supportive care needs. The needs of partners are strongly related to their own distress, anxiety and depression. Most needs are health system and information needs as well as psychological needs. For example, “Having one member of hospital staff with whom you can talk to about all aspects of condition, treatment and follow up” is frequently mentioned (38.2%) as a health system need, as “Being informed as soon as possible about cancer which is under control or diminishing” (46.1%) in relation to information-related needs. This order seems to be the same for patients with breast cancer (Fiszer et al., 2014). A study in the outpatient setting reports similarly high needs in family caregivers of patients with different cancer diagnoses, but in reverse order (Rosenberger et al., 2012). In this study the relatives showed particularly high needs with regard to psychological needs followed by health system and information needs. It should also be noted that the relatives in this sample were predominantly female (67%), which could also be the reason for this order. These findings underline the high and so far often neglected, mental burden of partners.

Considering the interaction of the couple with regard to perceived burden and coping, the systemic-transactional theory model (Bodenmann, 1997), provides a useful framework for the interpretation of our results. Both partners are affected by the cancer diagnosis and its treatment. Partners include the stress of the partner in their own actions. For example, an overestimation of the partner’s need for support by the patient (other reported assessment) could be an expression of a desire to restore or maintain the dyadic system. This desire could be described as a supportive coping by the couple (Bodenmann, 2000). Alternatively, coping could take place by transferring the perceived burden. A recent study in Germany showed that couples in whom one partner had been diagnosed with cancer used similar coping strategies (Osin et al., 2018).

High levels of distress might indicate the need for specific psycho-oncological support for women with breast cancer as well as for male partners. Despite existing evidence that in couples with highly distressed patients with breast cancer and their partners demonstrated, that both patients and partners benefit from strengths-based interventions (Brandão et al., 2013; Bitz et al., 2015) partners are often not integrated in the health care system and available support offers according to their needs. A meta-analysis, which could include 10 randomized studies with psychotherapeutic couple-based interventions, showed significant improvements in mental stress (Faller et al., 2013).

However, there is also some evidence that especially in couples with a high relationship functioning at baseline, a psychological intervention does provide significant benefit (Traa et al., 2015). A study examined different aspects of couples with one of them suffering from cancer (Magsamen-Conrad et al., 2015). Here communication efficacy proved to be as an effective predictor for coping. An study on a dyadic coping (Saita et al., 2016) could demonstrate that patients who participated in a Cancer Dyads Group Intervention benefited regarding to the fighting spirit. One aim of the intervention was the improvement of patient engagement and the promotion of the relationship between patients and their informal caregivers. Individuals in the intervention group showed changes in all coping styles evaluated. As a further development of our results a group intervention for couples according to the Cancer Dyads Group Intervention would also be conceivable.

Our study has some limitations. First, study design was cross-sectional and thus cannot provide long-term data or give an indication on cause and effect. Second, while the response rate of 68% was relatively high, the completer rate was, however only 41%. There is a risk of a sample bias and we do not know, if couples with high relationship satisfaction or couples with high levels of distress are overrepresented in the sample. Previous research suggests that especially couples who are able to express their fears, feelings and needs in connection with cancer-specific issues report a higher level of relationship functioning (Traa et al., 2015). Perhaps these were the very couples who participated in our study. Furthermore, we have not measured communication efficacy, which may play a major role in managing cancer (Magsamen-Conrad et al., 2015). Thus, generalization of the results is limited. Hypotheses for further studies can be derived from this. **For testing, patients with different tumor diagnoses and their partners should be selected.** This could help to answer the question as to how far the sex of the patient and the type of oncological treatment plays a role in the management of the disease. It is important to measure dyadic coping, ideally over the course of the disease. For this purpose we suggest a mixed-method study design. For example, interviews with patients and partners could complement quantitative results with even deeper insights. Distress screening is nowadays state of the art in the interdisciplinary care for cancer patients, as mandated by current treatment guidelines (2020). However, our findings indicate that in addition to that a systematic distress and needs assessment for male partners of patients with breast cancer should be routinely implemented. According to the results of the screening and the preferences of patients and partners, psycho-oncological support services should offer both individual and couple-based interventions.

## Data Availability Statement

The datasets for this article are not publicly available because of data protection principles. Requests to access the datasets should be directed to UG, ute.goerling@charite.de.

## Ethics Statement

The studies involving human participants were reviewed and approved by the Ethics Board Hamburg. The patients/participants provided their written informed consent to participate in this study.

## Author Contributions

UG analyzed the data and wrote the manuscript. CB co-wrote the manuscript. AM-T and VM planned the study, analyzed, and co-wrote the manuscript. All authors contributed to the article and approved the submitted version.

## Conflict of Interest

The authors declare that the research was conducted in the absence of any commercial or financial relationships that could be construed as a potential conflict of interest.
